# Supplementary material for: The RNA-Binding Landscape of HAX1 Protein Indicates Its Involvement in Translation and Ribosome Assembly
Source: Cells. 2022 Sep 20;11(19):2943. doi: 10.3390/cells11192943 (PMC9564044; doi:10.3390/cells11192943)
Supplement: Supplementary file 1 [file cells-11-02943-s001.zip › SUPPLEMENTAL DATA_corrected.pdf]

SUPPLEMENTAL DATA

Figures:

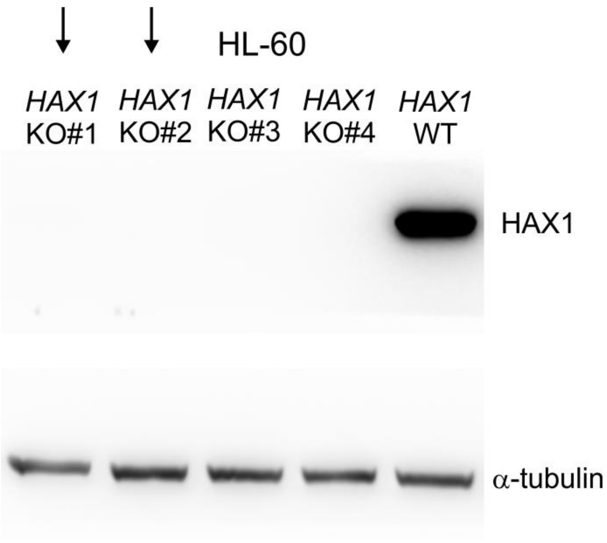

Figure S1. HAX1 protein is not present in *HAX1* KO cell lines used in analysis.

Western blot with four established *HAX1* KO cell lines (#1-4) and the wild type (HL-60 cell line). α-tubulin represents the reference. *HAX1* KO cell lines marked with arrows were used in RNA-seq and sedimentation experiments.

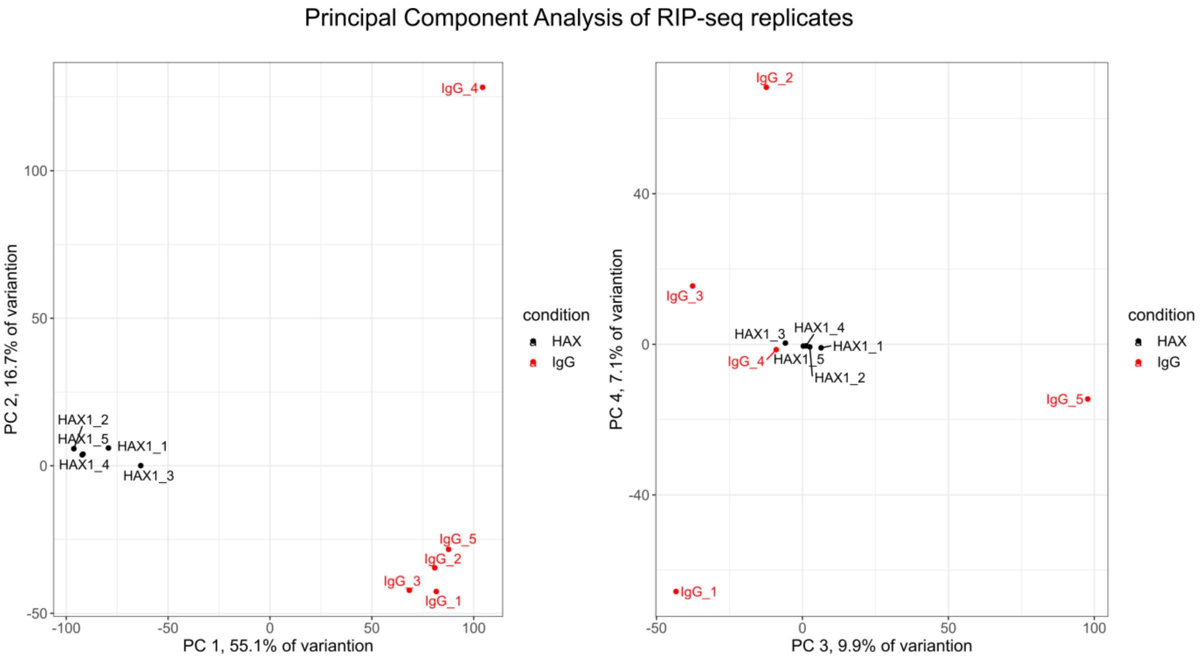

Figure S2. Principal Component Analysis of five RIP-seq replicates shows correct clustering of data.

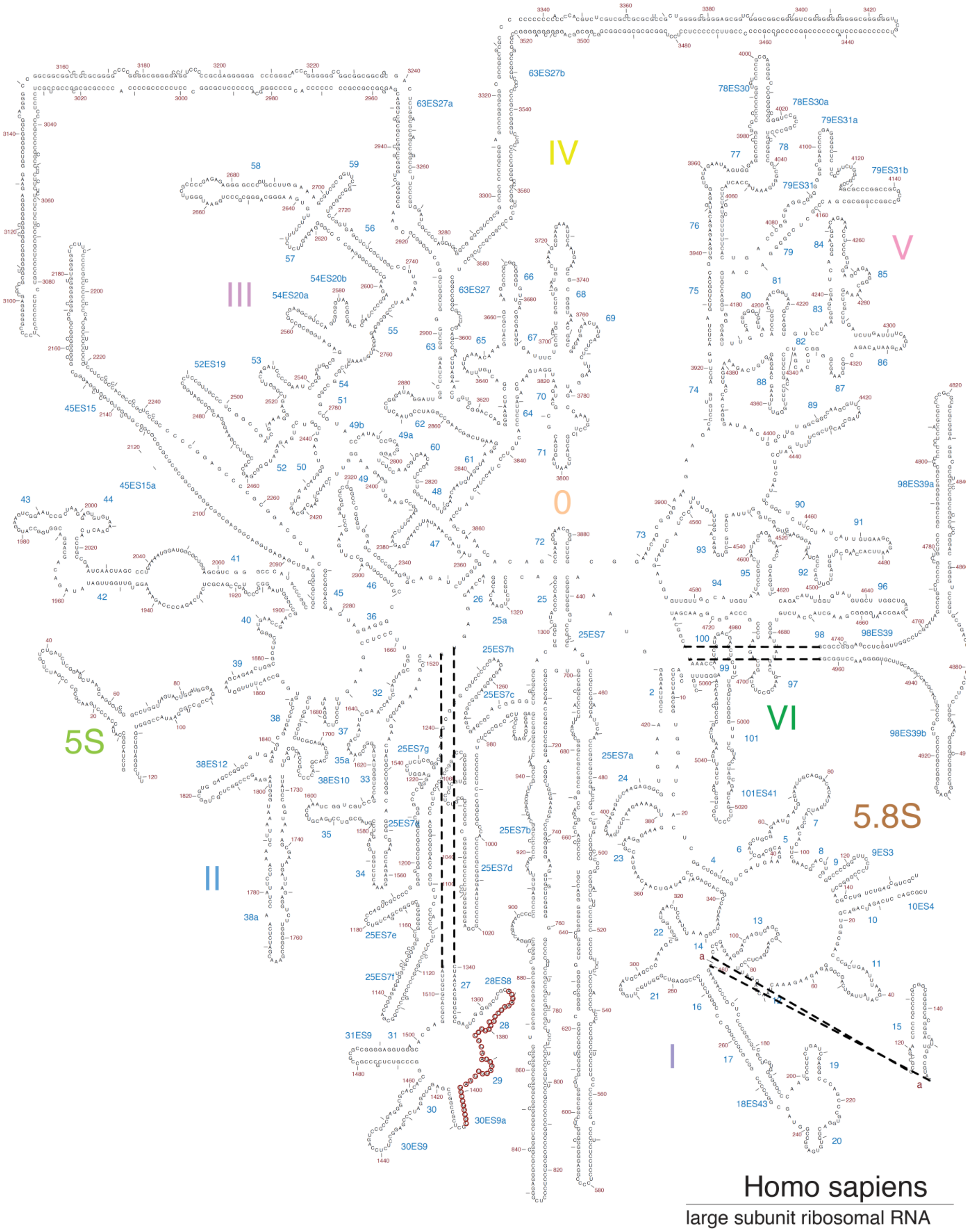

Figure S3. Large ribosomal subunit rRNA with marked region of potential interaction site with HAX1 (nucleotides marked by red circles).

A

B

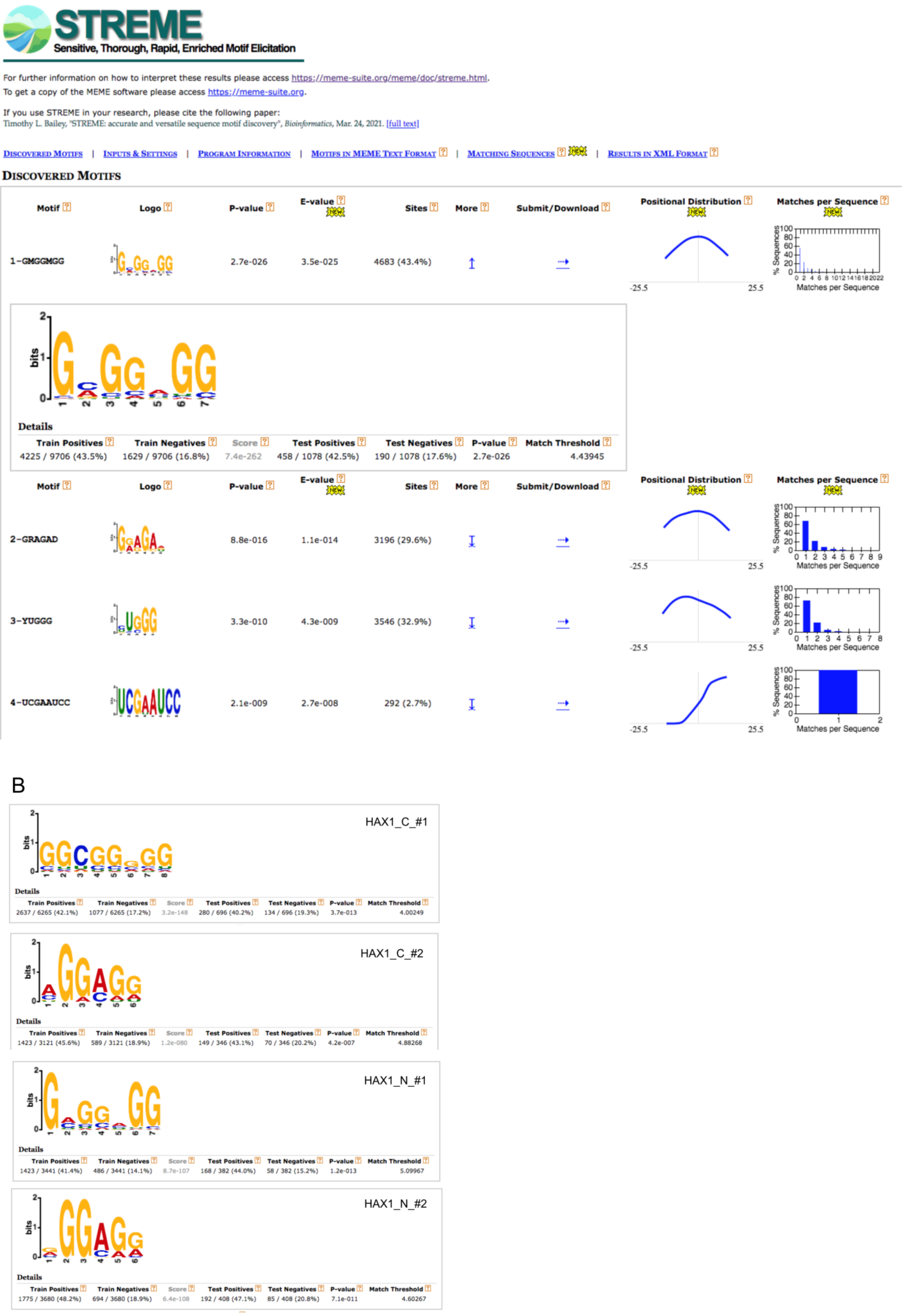

STREME
Sensitive, Thorough, Rapid, Enriched Motif Elicitation
For further information on how to interpret these results please access https://meme-suite.org/meme/doc/streme.html.
To get a copy of the MEME software please access https://meme-suite.org.
If you use STREME in your research, please cite the following paper:
Timothy L. Bailey, "STREME: accurate and versatile sequence motif discovery", Bioinformatics, Mar. 24, 2021. [full text]
Discovered Motifs | Inputs & Settings | Program Information | Motifs in MEME Text Format | Matching Sequences | Results in XML Format
Discovered Motifs
Motif Logo P-value E-value Sites More Submit/Download Positional Distribution Matches per Sequence
1-GMGGMGG 2.7e-026 3.5e-025 4683 (43.4%)
Details
Train Positives Train Negatives Score Test Positives Test Negatives P-value Match Threshold
4225 / 9706 (43.5%) 1629 / 9706 (16.8%) 7.4e-262 458 / 1078 (42.5%) 190 / 1078 (17.6%) 2.7e-026 4.43945
2-GRAGAD 8.8e-016 1.1e-014 3196 (29.6%)
3-YUGGG 3.3e-010 4.3e-009 3546 (32.9%)
4-UCGAAUCC 2.1e-009 2.7e-008 292 (2.7%)
-25.5
25.5
% Sequences
Matches per Sequence
bits
HAX1_C_#1
Details
Train Positives Train Negatives Score Test Positives Test Negatives P-value Match Threshold
2637 / 6265 (42.1%) 1077 / 6265 (17.2%) 3.2e-148 280 / 696 (40.2%) 134 / 696 (19.3%) 3.7e-013 4.00249
HAX1_C_#2
Details
Train Positives Train Negatives Score Test Positives Test Negatives P-value Match Threshold
1423 / 3121 (45.6%) 589 / 3121 (18.9%) 1.2e-080 149 / 346 (43.1%) 70 / 346 (20.2%) 4.2e-007 4.88268
HAX1_N_#1
Details
Train Positives Train Negatives Score Test Positives Test Negatives P-value Match Threshold
1423 / 3441 (41.4%) 486 / 3441 (14.1%) 8.7e-107 168 / 382 (44.0%) 58 / 382 (15.2%) 1.2e-013 5.09967
HAX1_N_#2
Details
Train Positives Train Negatives Score Test Positives Test Negatives P-value Match Threshold
1775 / 3680 (48.2%) 694 / 3680 (18.9%) 6.4e-108 192 / 408 (47.1%) 85 / 408 (20.8%) 7.1e-011 4.60267

Figure S4. STREME analysis of CRAC data.

A. STREME analysis of combined CRAC data for HAX1-binding motif in mRNA targets. B. Results of STREME analyses performed for separate replicates.

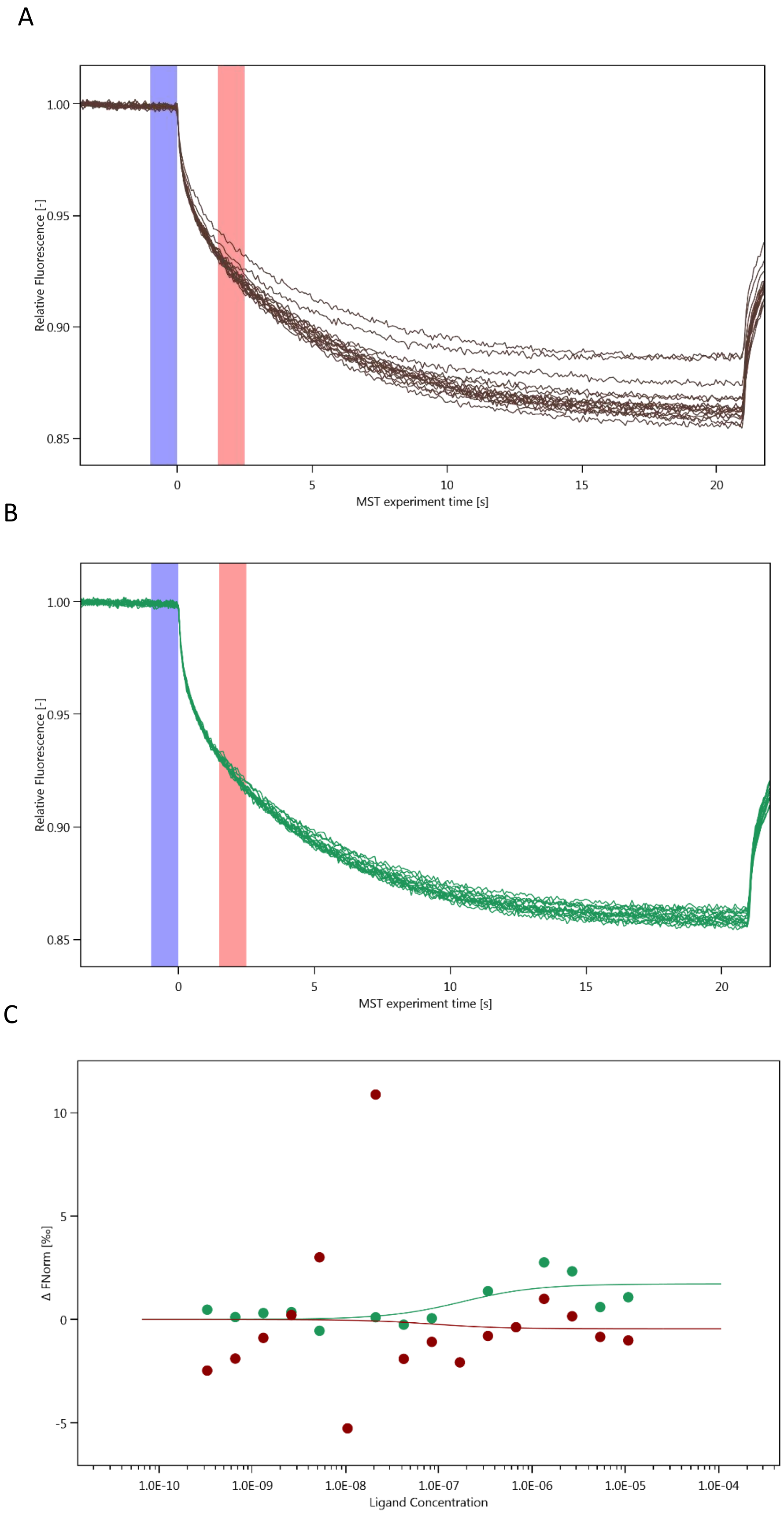

A
1.00
0.95
0.90
0.85
Relative Fluorescence [-]
0
5
10
15
20
MST experiment time [s]
B
1.00
0.95
0.90
0.85
Relative Fluorescence [-]
0
5
10
15
20
MST experiment time [s]
C
10
5
0
-5
Δ FNorm [‰]
1.0E-10
1.0E-09
1.0E-08
1.0E-07
1.0E-06
1.0E-05
1.0E-04
Ligand Concentration

Figure S5. The raw MST data for HAX1-RPL19mRNA interaction.

MST raw data of (A) antisense (*in vitro* transcript of the RPL19) in buffer as negative control of experiment, and (B) sense (*in vitro* transcript of the RPL19) in TRIS buffer binding to HAX1 protein. Sense RNA provides well-defined curves. The cold region is set to 0s (blue) and the hot region to 3s (red) to determine the K_d of the interaction and to avoid potential convection phenomena. (C) Dose-response curve for the binding interaction between HAX1 protein and RPL19 *in vitro* transcript (sense as green line, and antisense as dark red line) in TRIS buffer. The concentration of HAX1 protein is kept constant at 50 nM, while the transcript concentration varies from 10.8 µM to 13.5 nM (green). The binding curve yields a K_d of 0.238 µM in this measurements. The negative control did not produce a binding curve (dark red).

A

TF	RIP FC	RIP adjusted p-value	Enrichr Combined Score (ENCODE)
MYC	1,204498	3,54E-09	302,66
TAF1	1,482814	1,11E-15	109,54
YY1	2,049345	3,7E-106	86,95
E2F6	1,431905	2,19E-12	86,83
BRCA1	1,200453	1,15E-07	62,52
USF1	4,655167	7,3E-104	16,48
MYCBP	3,285746	3,1E-113	
MYCBP2	1,287111	1,29E-08	

B

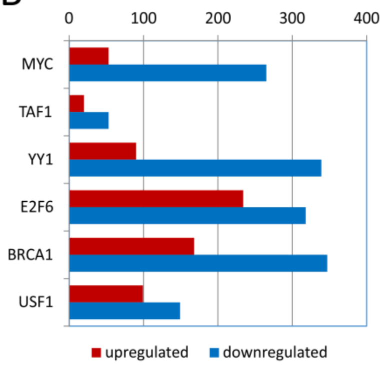

Figure S6. Analysis of transcription factors detected in RIP-seq selected as regulating transcripts differently expressed in *HAX1* KO.

A. Analysis of transcription factors possibly affected by *HAX1* KO. A. A list of the most probable transcription factors identified by Enrichr as regulating a subset of genes with significantly changed expression in *HAX1* KO simultaneously identified as RIP targets. The two MYC-binding proteins are included as high-scoring in RIP and with a potential of influencing the performance of MYC . B. A bar plot showing the number of upregulated and downregulated genes in *HAX1* KO for the specific TFs.

Supplementary files description (Excel files):

File S1. RIP-seq results; experimental data and analysis.

Sheet1 (all): results for all genes (pulldown: HAX1/IgG)

Sheet2 (positive): only positive targets with FC and FDR

Sheet3 (RNA classes): RNA biotypes detected in RIP

Sheet4 (DAVID_BP): enrichments in Biological Process for RIP targets (DAVID)

Sheet5 (Panther GO BP): enrichments in Slim Biological Process (Gene Ontology Panther)

File S2. CRAC results; experimental data and analysis.

Sheet1 (mRNA_counts_hOH7 FC): results for all genes (*HAX1* overexpressing cell line/control cell line)

Sheet2 (Gene_Symbol_N_C_overlap): targets overlapping in N and C CARC datasets

Sheet3 (RNA classes): RNA biotypes detected in CRAC

Sheet4 (Genomic position): genomic position of pooled CRAC targets

Sheet5 (DAVID_BP): enrichments in Biological Process for pooled CRAC targets

Sheet6 (Panther GO BP): enrichments in Slim Biological Process (Gene Ontology Panther) of pooled CRAC targets

File S3. Overlap of RIP and CRAC results

Sheet1 (Gene name): Lists of gene names representing significant results obtained in RIP and CRAC (HAX1_C) and the overlapping part

Sheet2 (DAVID_BP): enrichments in Biological Process for the overlapping RIP and CRAC targets.

Sheet5 (Panther GO BP): enrichments in Slim Biological Process (Gene Ontology Panther) for the overlapping RIP and CRAC targets.

File S4. RNA-seq (*HAX1* KO/WT) results.

Sheet1 (all): results for all genes, FC calculated as a combined value for HAX1 KO#1 and HAX1 KO#2

Sheet2 (downregulated): only significantly downregulated genes

Sheet3 (upregulated): only significantly upregulated genes

Sheet4 (GO_Biological Process_String11): String11 weighted analysis of the results with FC(RNA-seq *HAX1* KO/RNA-seq WT) as values.

File S5. Correlation of expression of *HAX1* with other genes in different neoplasm (cBioPortal analysis)

Sheet1 (InvBC_genes): Invasive Breast Cancer (TCGA, PanCancer Atlas), gene names and correlation assessment (Spearman coefficient, p-value, q-value)

Sheet2 (InvBC_BP): Invasive Breast Cancer, String 11 GO weighted analysis with Spearman coefficient as values, Biological Process terms

Sheet3 (Cervical_carcinoma_genes): Cervical Carcinoma (TCGA, PanCancer Atlas), gene names and correlation assessment (Spearman coefficient, p-value, q-value)

Sheet4 (Cervical Carcinoma_BP): Cervical Carcinoma, String 11 GO weighted analysis with Spearman coefficient as values, Biological Process terms

Sheet5 (AML_genes): Acute Myeloid Leukemia (TCGA, PanCancer Atlas), gene names and correlation assessment (Spearman coefficient, p-value, q-value)

Sheet6 (AML_BP): Acute Myeloid Leukemia, String 11 GO weighted analysis with Spearman coefficient as values, Biological Process terms

Sheet7 (DLBCL_genes): Diffuse Large B Cell Lymphoma (TCGA, PanCancer Atlas), gene names and correlation assessment (Spearman coefficient, p-value, q-value)

Sheet8 (DLBCL_BP): Diffuse Large B Cell Lymphoma, String 11 GO weighted analysis with Spearman coefficient as values, Biological Process terms
